# Enhancement of Matrix Metalloproteinase-2 (MMP-2) as a Potential Chondrogenic Marker during Chondrogenic Differentiation of Human Adipose-Derived Stem Cells

**DOI:** 10.3390/ijms17060963

**Published:** 2016-06-17

**Authors:** Yoshie Arai, Sunghyun Park, Bogyu Choi, Kyoung-Won Ko, Won Chul Choi, Joong-Myung Lee, Dong-Wook Han, Hun-Kuk Park, Inbo Han, Jong Hun Lee, Soo-Hong Lee

**Affiliations:** 1 Department of Biomedical Science, CHA University, Seongnam-si, Gyeonggi-do 443-742, Korea; ruddov@gmail.com (Y.A.); nightsky9836@naver.com (S.P.); bgchoi725@gmail.com (B.C.); glintk@naver.com (K.-W.K.); 2 Department of Orthopedic Surgery, Bundang Medical Center, CHA University, Seongnam-si, Gyeonggi-do 443-742, Korea; wcosdoc@gmail.com (W.C.C.); drjmlee@naver.com (J.-M.L.); 3 Department of Optics and Mechatronics Engineering, BK21+ Nano-Integrated Cogno-Mechatronics Engineering, College of Nanoscience & Nanotechnology, Pusan National University, Busan 619-961, Korea; nanohan@pusan.ac.kr; 4 Department of Biomedical Engineering, Collage of Medicine, Kyung Hee University, Seoul 151-742, Korea; sigmoidus@khu.ac.kr; 5 Department of Neurosurgery, Bundang Medical Center, CHA University, Seongnam-si, Gyeonggi-do 443-742, Korea; hanib@cha.ac.kr; 6 Department of Food Science and Biotechnology, College of Life Science, CHA University, Gyeonggi-do 443-742, Korea

**Keywords:** human adipose-derived stem cells, chondrogenic differentiation, extracellular matrix, matrix metalloproteinase-2

## Abstract

Human adipose-derived stem cells (hASCs) have a capacity to undergo adipogenic, chondrogenic, and osteogenic differentiation. Recently, hASCs were applied to various fields including cell therapy for tissue regeneration. However, it is hard to predict the direction of differentiation of hASCs in real-time. Matrix metalloproteinases (MMPs) are one family of proteolytic enzymes that plays a pivotal role in regulating the biology of stem cells. MMPs secreted by hASCs are expected to show different expression patterns depending on the differentiation state of hASCs because biological functions exhibit different patterns during the differentiation of stem cells. Here, we investigated proteolytic enzyme activity, especially MMP-2 activity, in hASCs during their differentiation. The activities of proteolytic enzymes and MMP-2 were higher during chondrogenic differentiation than during adipogenic and osteogenic differentiation. During chondrogenic differentiation, mRNA expression of MMP-2 and the level of the active form of MMP-2 were increased, which also correlated with Col II. It is concluded that proteolytic enzyme activity and the level of the active form of MMP-2 were increased during chondrogenic differentiation, which was accelerated in the presence of Col II protein. According to our findings, MMP-2 could be a candidate maker for real-time detection of chondrogenic differentiation of hASCs.

## 1. Introduction

Human adipose-derived stem cells (hASCs) are used for tissue regeneration because of their potential to undergo various types of differentiation, such as adipogenic, chondrogenic, and osteogenic differentiation [1,2]. However, like other types of stem cells, it is difficult to predict and control the direction of differentiation of hASCs in real-time. To evaluate the direction of differentiation of hASCs, many scientists have reported various differentiation markers of hASCs; for instance, peroxisome proliferator-activated receptor gamma, CCAAT/enhancer-binding protein beta, and adiponectin for adipogenic differentiation; type II collagen (Col II), sex determining region Y-box 9 (Sox9), and aggrecan for chondrogenic differentiation; and runt-related transcription factor 2 (RUNX2), alkaline phosphatase (ALP), and type I collagen (Col I) for osteogenic differentiation. By analyzing data obtained from PCR, Western blot, immunohistochemistry, and other analyses, the expression of these markers can be used to evaluate the differentiation state of hASCs. However, it is still hard to investigate the differentiation potential of hASCs in real-time during differentiation because these analytical methods damage live cells through the isolation of DNA and proteins and, furthermore, the analysis takes time. Thus, it is very critical to evaluate the differentiation of live hASCs in real-time to prepare therapeutic hASCs for clinical applications.

Proteolytic enzymes, also called proteinases or peptidases, are essential for stem cell development [3]. They have a major role in the cleavage of specific peptide bonds. Matrix metalloproteinases (MMPs) are one family of proteolytic enzymes. There are more than 25 members of the MMP family in mammals and they are classified as collagenases (MMP-1, -8, and -13), gelatinases (MMP-2 and -9), and stromelysins (MMP-3, -10, and -11) according to their enzyme-substrate specificity [4,5]. They are also classified as secreted and membrane-tethered MMPs (MMP-14, -15, -16, and -17). Among MMP family members, MMP-2 remodels the extracellular matrix (ECM) and activates growth factors, which are crucial events during the proliferation, migration, and differentiation of cells [3,5,6,7]. Furthermore, MMP-2 expression is increased during the development of rodents, and MMP-2-deficient mice show defects in cartilage formation [8,9]. Based on these reports, we reason that maintenance of MMP-2 expression is required for the chondrogenic differentiation of hASCs.

On the other hand, Col II is a major ECM component of joint cartilage, which can be cleaved and remodeled by MMP-2. Bosnakovski *et al*. reported that Col II stimulates the chondrogenic differentiation of mesenchymal stem cells (MSCs) and the effect of transforming growth factor (TGF)-β1 on MSCs [10]. However, there are a few studies reporting a correlation between MMP-2 and differentiation of hASCs grown on Col II.

In this study, we hypothesized that proteolytic enzymes including MMP-2 have a specific expression pattern depending on the differentiation state (adipogenic, chondrogenic, and osteogenic differentiation) and that ECM (Col II) increases MMP-2 activity during chondrogenic differentiation. To verify the hypothesis, we examined the proliferation and migration of hASCs during differentiation. We investigated the activities of enzymes and secreted MMP-2 during differentiation of hASCs. In addition, the effects of Col II on MMP-2 expression patterns during the chondrogenic differentiation of hASCs were examined. Thus, the purpose of our study was to predict chondrogenic differentiation by observing the MMP-2 expression pattern. Our findings could represent a milestone by providing a powerful means to observe chondrogenic differentiation of hASCs in real-time.

## 2. Results

### 2.1. Characterization of hASCs

**Figure 1:**
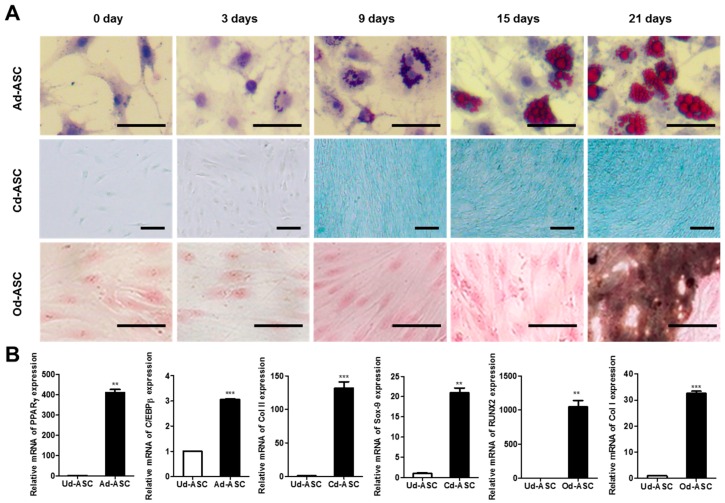

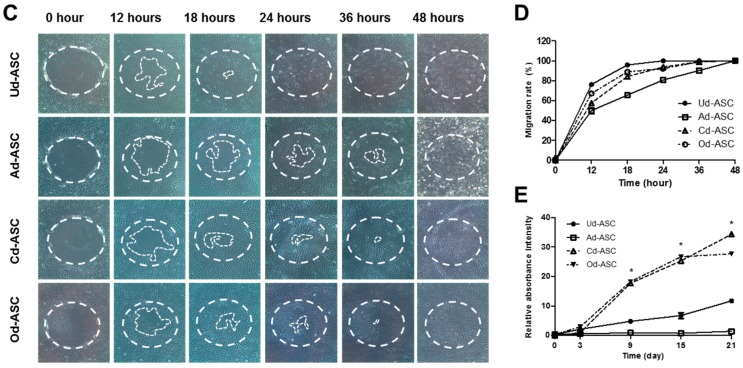
Characterization of hASCs. (**A**) hASCs were incubated with adipogenic, chondrogenic, and osteogenic differentiation medium for 21 days. The efficiencies of differentiation were determined by histological staining as Oil Red O staining, Alcian Blue staining, and von Kossa staining, respectively (scale bar = 50 μm); (**B**) the mRNA expression of differentiation marker was measured by qPCR after 21 days of induction. The data are shown as the mean ± SEM (*n* = 3, **: *p* < 0. 005, ***: *p* < 0.0005); (**C**) the migration of Ud-ASC, Ad-ASC, Cd-ASC, and Od-ASC were investigated using Radius™ 24-Well Cell Migration Assay. The broken line circles indicate the rim of the cells prior to migration; (**D**) the quantitative data of the migration area was calculated by ImageJ software (*n* = 3); and (**E**) the proliferation of Ud-ASC, Ad-ASC, Cd-ASC, and Od-ASC was measured using CCK-8 kit. The data are shown as the mean ± SEM (*n* = 3, Ud-ASC *vs.* Cd-ASC, *: *p* < 0. 05).

To examine the multipotency of hASCs, three types of differentiation such as adipogenic, chondrogenic, and osteogenic differentiation of hASCs were induced for 21 days with adipogenic medium (AM), chondrogenic medium (CM), and osteogenic medium (OM), respectively. Staining for markers of each type of differentiation indicated the multipotent capacity of hASCs (Figure 1A). These results indicate the evidence for multipotency of hASCs. Lipid accumulation and GAG contents were observed after nine days with AM and CM treatment, respectively. Whereas, mineralization was detected after 21 days in long-term induction with OM. Typically, it is known that lipid and GAG accumulation of hASCs are observed after 7–14 days of adipogenic and chondrogenic differentiation, and mineralization is observed after 14–21 days of osteogenic differentiation [11,12,13]. In addition, mRNA level of tri-lineage differentiation marker genes was investigated by qPCR after 21 days of induction (Figure 1B). The mRNA of *PPARγ* and *C/EBPβ*, as adipogenic marker, in hASCs undergoing adipogenic differentiation (Ad-ASC) were 410-fold and three-fold higher than that of undifferentiating hASCs (Ud-ASC). The mRNA of *Col II* and *SOX9*, as a chondrogenic marker, in hASCs undergoing chondrogenic differentiation (Cd-ASC) were 132-fold and 21-fold higher than that of Ud-ASC. The mRNA of *RUNX2* and *COL I*, as an osteogenic marker, in hASCs undergoing osteogenic differentiation (Od-ASC) were 1045-fold and 33-fold higher than that of Ud-ASC. Proliferation of hASCs during differentiation was calculated by CCK-8 assay (Figure 1E). The proliferation of Ad-ASC in AM was 8.3-fold lower than Ud-ASC in growth medium after 21 days. The proliferation of Cd-ASCs (2.9 to 3.7-fold increase) and Od-ASCs (2.4- to 4.0-fold increase) was much higher after nine days of differentiation induction than that of Ud-ASCs. After 21 days of induction, the proliferation of Cd-ASCs was 1.2-fold higher than that of Od-ASCs. The migration of hASCs with growth medium, AM, CM, or OM was observed for 48 h using the Radius™ 24-Well Cell Migration assay (Figure 1C,D). The migration rate of Ad-ASC, Cd-ASC, and Od-ASC at 12 h was 1.5-, 1.3-, and 1.1-fold decreased than that of Ud-ASC. After 18 h, the migration rate of Cd-ASC and Od-ASC showed similar motility.

### 2.2. Proteolytic Enzyme Activity in hASCs during Differentiation

**Figure 2:**
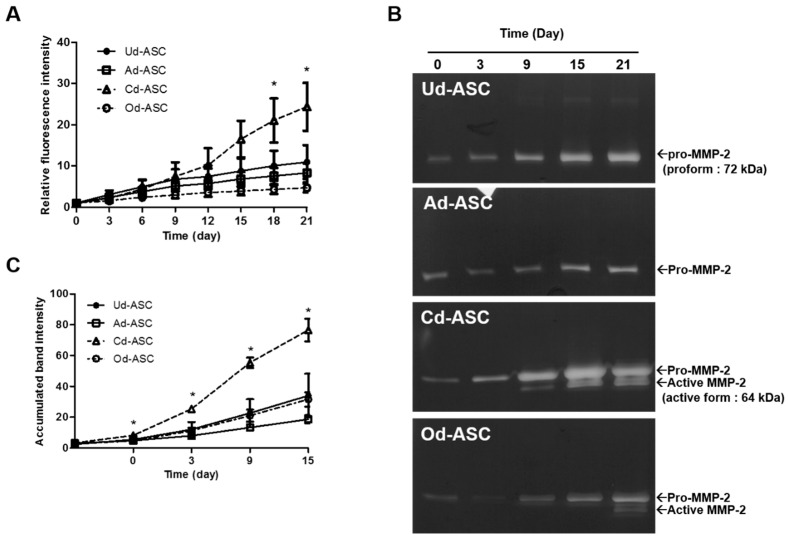
Proteolytic enzyme activity in hASCs during differentiation. (**A**) The proteolytic enzyme activities of Ud-ASC, Ad-ASC, Cd-ASC, and Od-ASC were detected by DQ-BSA; (**B**) the MMP-2 activities of Ud-ASC, Ad-ASC, Cd-ASC, and Od-ASC were measured by zymography. The molecular weight of MMP-2 pro-form is 72 kDa and the active form is 62 kDa; and (**C**) the quantitative data of accumulated band intensity of MMP-2, both pro-form and active-form. The band intensity (in panel **B**) was measured by ImageJ software. The data are shown as the mean ± SEM (*n* = 3, Ud-ASC *vs.* Cd-ASC, *: *p* < 0.05).

It is known that various proteolytic enzymes play an important role for differentiation of hASCs. In this study, DQ-BSA was used to quantify proteolytic enzyme activity. In the presence of proteolytic enzymes such as collagenase, DQ-BSA was cleaved and produced a green fluorescence. The fluorescence intensity was measured by fluorescence reader. During differentiation of hASCs, proteolytic enzyme activities were evaluated by fluorescence intensity generated from DQ-BSA (Figure 2A). Cd-ASC showed a time-dependent increase in proteolytic enzyme activity and much higher proteolytic enzyme activity compared to Ud-ASC, Ad-ASC and Od-ASC after 18 days of induction. Additionally, to demonstrate the relationship between the chondrogenic differentiation and MMP-2 in Cd-ASC, the activity of MMP-2 was observed using gelatin zymography (Figure 2B,C). The prominent band of pro- (72 kDa) and active-form (62 kDa) of MMP-2 can be detected by gelatin zymography [14]. MMP-2 activity of hASCs was increased depending on culture time. Interestingly, in Cd-ASC, the higher level of MMP-2 expression was found compared to Ud-ASC, Ad-ASC and Od-ASC. In addition, active-form of MMP-2 was accumulated after nine days after differentiation in Cd-ASC, it is a relatively short time point compared to Od-ASC. On the other side, it was not observed that active-form of MMP-2 in Ud-ASC and Ad-ASC. Taken together with those results, it is demonstrated that CM strongly stimulate MMP-2 activity of hASC during differentiation.

### 2.3. Chondrogenic Differentiation of hASCs on Col II-Coated Plates

**Figure 3:**
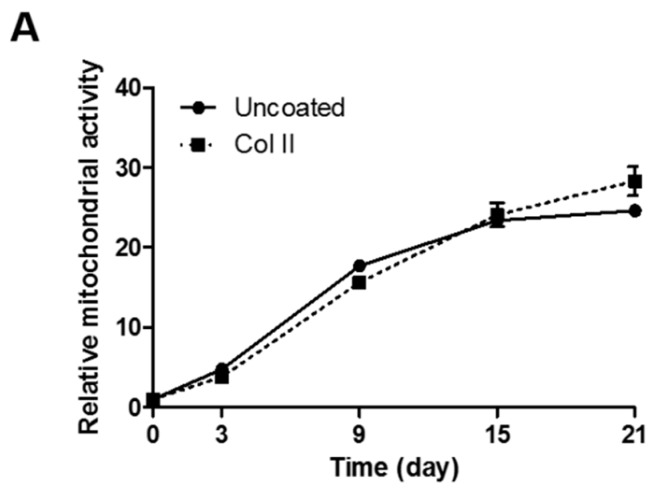

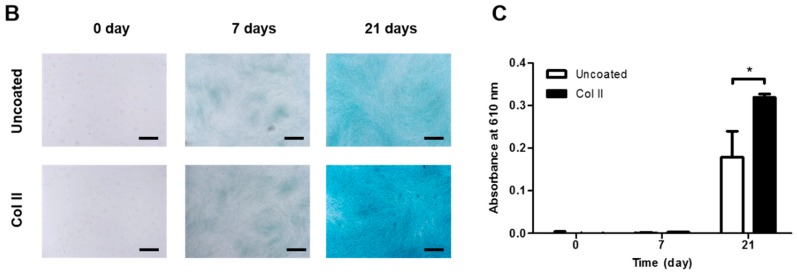
Priming effect of Col II for chondrogenic differentiation of hASCs. (**A**) The proliferation of Cd-ASC was determined on normal and Col II-coated plates by CCK-8 assay. The data are shown as the mean ± SEM (*n* = 3, ns: *p* = 0. 3922); (**B**) the chondrogenic differentiation was confirmed by Alcian Blue staining after 0, 7, and 21 days of induction (scale bar = 500 μm); and (**C**) the GAG contents were assessed by Alcian Blue extraction with 8 M of guanidine hydrogen chloride. The absorbance was measured at 610 nm by microplate reader. The data are shown as the mean ± SEM (*n* = 3, *: *p* < 0.05).

It is well known that type II collagen (Col II) is major component of extracellular matrix in cartilage and it can enhance the chondrogenic differentiation of hASCs. The effect of Col II on proliferation of Cd-ASC was investigated on normal and Col II-coated culture plates (Figure 3A). There was no significant difference between two curves of proliferation of Cd-ASC on normal and Col II-coated plates (two-way ANOVA, *p* = 0. 3922). On the other hand, priming effect of Col II on chondrogenic differentiation results in significantly higher expression of GAG contents on Col II-coated plates compared with normal plates, which is confirmed by Alcian Blue staining (Figure 3B,C). These illustrates that Col II protein is able to influence only chondrogenic differentiation of hASCs, not proliferation.

### 2.4. MMP-2 and Hypertrophic Marker Genes Expression of hASCs during Chondrogenic Differentiation in the Presence of Col II

**Figure 4:**
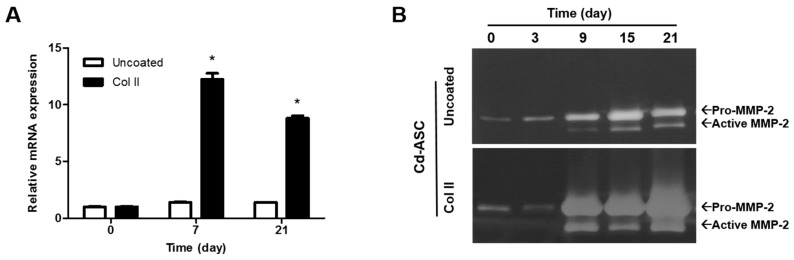

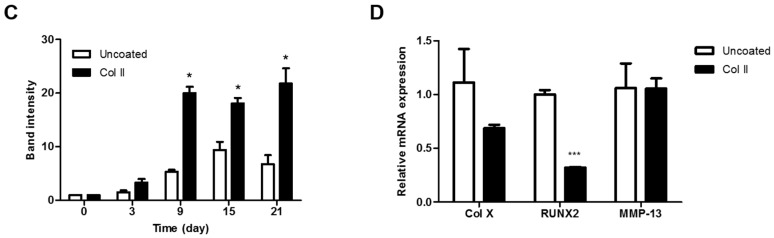
MMP-2 activity in hASCs during chondrogenic differentiation on Col II-coated plates. (**A**) The mRNA level of MMP-2 of Cd-ASC was determined on normal and Col II-coated plates by qPCR; (**B**) the MMP-2 activities of Cd-ASC were measured on normal plates and Col II-coated plates by zymography; (**C**) the quantitative data of MMP-2 band intensity (in panel B) was measured using ImageJ software; and (**D**) the expression of hypertrophic marker genes, *COL X*, *RUNX2*, and *MMP-13*, was investigated by qPCR. The data are shown as the mean ± SEM (*n* = 3, *: *p* < 0.05, ***: *p* < 0.0005).

To explore the expression of MMP-2, the mRNA level and the protein level were quantified by real-time PCR and zymography (Figure 4A,B). The band intensity in zymography was quantified using ImageJ software (NIH, Bethesda, MD, USA) (Figure 4C). The mRNA expression of Cd-ASC on Col II-coated plate was significantly increased compared to Cd-ASC on normal plate after 7 and 21 days, which indicated that the presence of Col II increased the *MMP-2* expression (Figure 4A). Moreover, the total protein level of MMP-2, both of pro- and active form, was increased approximately 1.9- to 3.8-fold in Cd-ASC on Col II-coated plate compared with that on normal plate as induction time advanced from nine days of culture (Figure 4B,C). It was demonstrated that the higher mRNA expression and activity of MMP-2 were maintained by the presence of Col II during chondrogenic differentiation. Hypertrophic marker genes (*COL X*, *RUNX2*, and *MMP-13*) of Cd-ASC were also determined by real-time PCR after 21 days of chondrogenic differentiation (Figure 4D). There were no significant differences between Cd-ASC on normal and Col II-coated plates in *COL X* and *MMP-13* expression. *RUNX2* expression of Cd-ASC was decreased on Col II-coated plates compared to on normal plates. This result implies that MMP-2 expression is not related to hypertrophy during chondrogenic differentiation.

## 3. Discussion

In this study, hASCs were used for all experiments as MSCs, which have the potential to undergo tri-lineage (adipogenic, chondrogenic, and osteogenic) differentiation. hASCs are isolated from adipose tissues, such as joint fat pads. Recently, many researchers have focused on tissue regeneration using hASCs as a cell source; however, many parts of the differentiation mechanism of hASCs are not well known.

Proliferation and migration are required in the development and differentiation of stem cells; it is important to generate sufficient functional cells and to arrange cells in suitable locations. Several reports demonstrated the effect of differentiation medium on the proliferation and migration of MSCs. Supplemental reagents in chondrogenic or osteogenic differentiation medium, such as dexamethasone (DEX), ascorbic acid, and TGF-β, stimulate differentiation and proliferation [15,16,17]. The differentiation and proliferation of MSCs are regulated not only by these supplemental reagents but also by the microenvironment including ECM and proteolytic enzymes [18,19,20,21]. For example, ECM deposited by adipose-derived stem cells (ASCs) and synovium-derived stem cells of mini-pigs improves the proliferation and chondrogenic differentiation of ASCs through reducing reactive oxygen species production [22]. In particular, there is much evidence that Col I and Col II, which are major ECM components in bone and cartilage, respectively, increase osteogenic and chondrogenic differentiation and proliferation via activation of focal adhesion proteins [23,24,25]. The priming effect of ECM during stem cell differentiation is closely related to ECM remodeling by proteolytic enzymes such as elastase, cathepsin G, and various MMPs [26,27]. Proteolytic enzymes play an important role in the biology of stem cells, such as their proliferation, migration, and differentiation [26,28]. Taken together, this could suggest that the pattern of proteolytic enzyme activities represents the type of differentiation (adipogenic, chondrogenic, and osteogenic).

MMPs are one proteolytic enzyme family. Although it is not clear whether MMPs are directly related to developmental processes, ECM remodeling by MMPs is a necessary process during embryonic development [5]. In cartilage development, it was reported that MMP activity is elevated during cellular condensation and ECM accumulation of mesenchymal precursor cells [29]. In addition, key signaling factors in cartilage development, fibroblast growth factor, and TGF-β are modulated by MMPs [5,30]. Among MMP family members, MMP-2 (also called gelatinase A and type IV collagenase) has a crucial role in ECM degradation followed by ECM remodeling [5,26]. Several studies reported that MMP-2 expression is increased in cartilage of mice during aging and is a negative regulator of the condensation of chick leg bud mesenchymal cells [31,32]. However, cartilage formation is attenuated by MMP-2 defection during the development of mice [8]. Although MMP-2 appears to play a pivotal role in cartilage tissue formation, the effect of MMP-2 is still unclear and has rarely been studied during chondrogenic differentiation of ASCs. Thus, in the present study, we sought to investigate and elucidate the activities of proteolytic enzymes and MMP-2 during chondrogenic differentiation of hASCs.

First, to investigate the nature of each type of differentiation of hASCs, we observed the proliferation and migration of undifferentiating hASCs (Ud-ASCs), hASCs undergoing adipogenic differentiation (Ad-ASCs), hASCs undergoing chondrogenic differentiation (Cd-ASCs), and hASCs undergoing osteogenic differentiation (Od-ASCs) in growth medium, adipogenic medium (AM), chondrogenic medium (CM), and osteogenic medium (OM), respectively (Figure 1). We found that there are different proliferation capacities of these cells, and assumed that the differentiation medium influences not only the direction of differentiation, but also the proliferation capacity (Figure 1A,E). Next, we investigated whether migration is associated with proliferation during the differentiation of stem cells. The migration of Cd-ASCs and Od-ASCs with higher proliferation was slower than that of Ud-ASCs with lower proliferation (Figure 1C–E). A few studies were reported that MMP-2 implicates in the proliferation and migration [32,33]. However, in the present study, there is no consistency between MMP-2 expression and the rates of proliferation or migration during differentiation of hASCs. In this study, each type of differentiation medium, which are widely used in most laboratories, was prepared based on the protocol provided by the manufacturer [11,34,35]. A relatively high concentration (1 μM) of DEX was included in AM, while a lower concentration (100 nM) was included in CM and OM. Several studies reported that a high concentration of DEX has a negative effect on proliferation, whereas a low concentration of DEX (~100 nM) has a positive effect on proliferation [36,37,38]. Our finding that Cd-ASCs and Od-ASCs, which were exposed to a lower concentration of DEX, exhibited increased proliferation is coincident with previous reports. Another important component of the medium is ascorbic acid, which is only included in CM and OM. Choi et al. reported that 250 μM ascorbic acid increases bone marrow-derived MSC proliferation [15].

Second, to investigate the regulation of ECM remodeling enzymes during the differentiation of hASCs, we measured proteolytic enzyme activities using the DQ-BSA assay (Figure 2). Despite the similar proliferation capacity of Cd-ASCs and Od-ASCs, proteolytic enzyme activity at 21 days was 5.2-fold higher in Cd-ASCs (Figure 2A). Moreover, MMP-2 activity was 2.4-fold higher in Cd-ASCs at 21 days than in Od-ASCs. This implies that the activities of proteolytic enzymes and MMP-2 during the differentiation of hASCs are dependent on the differentiation medium and not on the rate of proliferation.

Lastly, we investigated MMP-2 expression in Cd-ASCs grown on Col II (Figure 3 and Figure 4), which increases chondrogenic differentiation of MSCs [39,40]. MMP-2 plays a role in Col II remodeling that would be able to regulate chondrogenic differentiation. While Col II enhanced the differentiation of Cd-ASCs, it did not change their proliferation (Figure 3A). However, MMP-2 activity in Cd-ASCs was increased when Col II was applied. This demonstrates that Col II is able to stimulate MMP-2 activity in Cd-ASCs, resulting in chondrogenic differentiation through ECM remodeling. Hypertrophy causes apoptosis, vascular invasion, and ossification of tissue; therefore, hypertrophy must be inhibited in MSC chondrogenesis [41,42]. We also investigated whether MMP-2 influences hypertrophy after 21 days of chondrogenic differentiation (Figure 4D). Leyh *et al*. reported that MMP-2 expression is increased in interleukin-1β-induced hypertrophic bone marrow MSCs [43]. However, in the present study, the expression levels of hypertrophic marker genes such as collagen type X and MMP-13 were the same in cells grown on normal plates and cells grown on Col II-coated plates, and expression of another marker, RUNX2, was decreased in the presence of Col II (Figure 4D). Thus, we suggested that MMP-2 mediates chondrogenic differentiation through Col II remodeling without induction of hypertrophy. In further study, we will identify the correlation between MMP-2 and chondrogenic markers (Sox9, aggrecan, and chondroitin sulfate), and investigate the remodeling of other ECMs (collagen type I, gelatin, and fibronectin) by MMP-2.

Several studies reported that the differentiation potential of hASCs is donor-dependent and unpredictable [44,45]. As previously mentioned, it is hard to control the direction of differentiation of multipotent MSCs. Thus, real-time monitoring of the direction of differentiation could help to guide differentiation and to control the differentiation fate of MSCs. Based on the present study, we proposed that chondrogenic differentiation of hASCs can be predicted through real-time monitoring of enzyme activity and MMP-2.

## 4. Materials and Methods

### 4.1. Isolation and Culture of hASCs

hASCs were isolated from adipose tissues in the articular fat pads around the knee. The adipose tissues were washed with phosphate-buffered saline (PBS) containing 2% (*v*/*v*) penicillin/streptomycin and digested by 0.5 mg/mL of collagenase in Dulbecco’s modified Eagle’s medium with low glucose (DMEM/LOW GLUCOSE, Hyclone, Logan, UT, USA) for 40 min. Digested adipose tissues were collected in 50 mL conical tube and centrifuged at 1000× *g* for 10 min. After centrifugation, supernatant fat was discarded and infranatant fluid was filtered using a 40 μm pore size of strainer. Filtered fluid was washed three times with DMEM by centrifugation at 1000× *g* for 10 min. Cell pellet was resuspended with growth medium (DMEM/LOW GLUCOSE containing 10% (*v*/*v*) fetal bovine serum (FBS) and 1% (*v*/*v*) penicillin/streptomycin (Hyclone, Logan, UT, USA) and cultured on tissue culture plate at 37 °C in 5% CO_2_ incubator. Medium was exchanged with fresh growth medium every three days. To investigate the chondrogenic differentiation on type II collagen (Col II), tissue culture plates were coated with 50 μg/mL of Col II at 4 °C overnight.

### 4.2. Differentiation of hASCs

hASCs were seeded on tissue culture plate at a cell density of 2 × 10^4^ cells/cm^2^ for adipogenic and osteogenic differentiation and 2 × 10^3^ cells/cm^2^ for chondrogenic differntiation. For adipogenic, chondrogenic, and osteogenic differentiation, hASCs incubated with adipogenic medium (AM), chondrogenic medium (CM), and osteogenic medium (OM). The composition of AM; high glucose medium (DMEM/HIGH GLUCOSE supplemented with 10% (*v*/*v*) FBS and 1% (*v*/*v*) penicillin/streptomycin), 10 μg/mL of insulin, 500 μM of 3-isobutyl-1-methylxanthine, 200 μM of indomethacin, 1 μM of dexamethasone. The composition of CM; high glucose medium, 1% (*v*/*v*) insulin transferrin selenium A (Gibco, Grand Island, NY, USA), 50 μg/mL of ascorbic acid, 100 nM of dexamethasone, 10 ng/mL of TGF-β1. The composition of OM; high glucose medium, 10 mM of glycerol-2-phosphate, 50 μg/mL of ascorbic acid, 100 nM of dexamethasone, GlutaMAX^TM^ (Gibco, Grand Island, NY, USA). The differentiation induced hASCs were incubated at 37 °C in 5% CO_2_ incubator, and medium was replaced every three days. The adipogenic, condrogenic, and osteogenic differentiation of hASCs was confirmed by Oil Red O, Alcian Blue, and von Kossa staining, respectively. Oil Red O staining was performed with hematoxylin as a counterstain. For the quantification of Alcian Blue stained glycosaminoglycans (GAGs), Alcian Blue was extracted with 8 M guanidine hydrogen chloride and the absorbance was measured at 610 nm using microplate reader.

### 4.3. Proliferation and Migration of hASCs

To investigate proliferation and migration during differentiation, hASCs were cultured at a cell density of 5 × 10^3^ cells/cm^2^. The rate of proliferation was determined using cell counting kit-8 (CCK-8, Dojindo, Japan) which can detect the mitochondrial activity. 20 μL of CCK-8 solution was treated in 200 μL of medium on hASCs undergoing differentiation. After 2 h, the absorbance of medium was measured at 450 nm using microplate reader (Molecular Devices, Sunnyvale, CA, USA). Migration of hASCs was investigated using Radius™ 24-Well Cell Migration Assay (Cell Biolabs, San Diego, CA, USA). Quantitative data of migration was analyzed using ImageJ software (NIH, Bethesda, MD, USA).

### 4.4. Measurement of Proteolytic Enzyme Activity

Proteolytic enzyme activity of hASCs during differentiation was measured using the DQ-BSA assay (dye quenched-bovine serum albumin, Molecular Probes, Eugene, OR, USA). Fluorescence was appeared by proteolytic cleavage of DQ-BSA. 1 mg/mL of DQ-BSA was coated on tissue culture plates at 4 °C for overnight. hASCs were cultured on DQ-BSA coated plates with AM, CM, and OM. For 21 days, fluorescence intensity caused by proteolytic enzyme activity of differentiated hASCs was measured at excitation 505 nm and emission 515 nm by fluorescence reader (TECAN, Männedorf, Switzerland).

### 4.5. Zymography

Serum-free medium was added on differentiation-induced hASCs, prior to collection of secreted proteins. After 24 h of incubation, medium was collected and mixed with non-reducing sodium dodecyl sulfate (SDS) sample buffer. Secreted proteins were separated by 10% of SDS-polyacrylamide gel containing 0.5% of gelatin. After electrophoresis, the gels were washed with renaturing buffer (20% (*v*/*v*) of Triton X-100 in DW) at room temperature for 30 min and incubated with developing buffer (50 mM of Tris-HCl (pH 7.5), 0.15 mM of NaCl, 5 mM of CaCl_2_, and 0.2% (*v*/*v*) of Brij^®^ 35 (Sigma-Aldrich, St. Louis, MO, USA) in DW at 37 °C for overnight. Developing buffer was discarded and Coomassie Blue staining solution (Bio-Rad, Hercules, CA, USA) was added on the gels. After 30 min of staining, destaining the gel by soaking for at least 2 h in destaining solution (45% of methyl alcohol and 10% of acetic acid in DW) with at least two changes of the solution until the background is nearly clear.

### 4.6. Quantitative Real-Time PCR

mRNA from undifferentiating hASCs or hASCs undergoing chondrogenic differentiation was extracted by Trizol™ (Life Technologies, Gaithersburg, MD, USA). cDNA was synthesized by TAKARA cDNA synthesis kit (TAKARA, Shiga, Japan), and then quantitative real-time PCR was performed using Power SYBR^®^ Green PCR Master Mix (Applied Biosystems, Warrington, UK). Primer pairs for *PPARγ*, *C/EBPβ*, *COL II*, *SOX9*, *COL I*, *COL X*, *MMP-13*, and *ribosomal protein S18* (*RPS18*) are shown in Table 1. *RPS18* gene was used as a housekeeping gene to normalize gene expression in for real-time PCR analysis.

### 4.7. Statistics

All statistical analysis were performed with GraphPad Prism ver.5.0 (GraphPad software, San Diego, CA, USA). *t*-test, a two-way ANOVA with Bonferroni post-test, or a one-way ANOVA with Tukey’s multiple comparison post-test was performed to compare the samples. Statistical significance was set at *p* < 0.05.

## 5. Conclusions

The proliferation of hASCs was much higher in CM than in other types of medium (growth medium, AM, and OM). Proteolytic enzyme and MMP-2 activities in hASCs were also increased during chondrogenic differentiation, and they were significantly higher during chondrogenic differentiation than during adipogenic or osteogenic differentiation. In the presence of Col II protein, which enhances chondrogenic differentiation, MMP-2 activity in hASCs was also increased. It is concluded that the activities of proteolytic enzymes and MMP-2 increase during chondrogenic differentiation, especially in the presence of Col II protein, which stimulates MMP-2. Based on our findings, we believe that MMP-2 is a potential real-time marker to evaluate chondrogenic differentiation.

## Figures and Tables

**Table 1:** Nucleotide sequences of primer pairs for real-time PCR.

Gene	Human Primer Sequence	
*PPARγ*	Sense	5′-GATACACTGTCTGCAAACATATCACAA-3′
	Antisense	5′-CCACGGAGCTGATCCCAA-3′
*C/EBPβ*	Sense	5′-GCAAGAGCCGCGACAAG-3′
	Antisense	5′-GGCTCGGGCAGCTGCTT-3′
*COL II*	Sense	5′-CACGTACACTGCCCTGAAGGA-3′
	Antisense	5′-CGATAACAGTCTTGCCCCACTT-3′
*SOX9*	Sense	5′-CCCCAACAGATCGCCTACAG-3′
	Antisense	5′-GAGTTCTGGTCGGTGTAGTC-3′
*RUNX2*	Sense	5′-CAGACCAGCAGCACTCCATA-3′
	Antisense	5′-CAGCGTCAACACCATCATTC-3′
*COL I*	Sense	5′-CCCCTGGAAAGAATGGAGATG-3′
	Antisense	5′-TCCAAACCACTGAAACCTCTG-3′
*COL X*	Sense	5′-ACGCTGAACGATACCAAATG-3′
	Antisense	5′-TGCTATACCTTTACTCTTTATGGTGTA-3′
*MMP-13*	Sense	5′-AACGCCAGACAAATGTGACC-3′
	Antisense	5′-AGGTCATGAGAAGGGTGCTC-3′
*RPS18*	Sense	5′-CTTCCACAGGAGGCCTACAC-3′
	Antisense	5′-CGCAAAATATGCTGGAACTTT-3′

## Abbreviations

Ad-ASC	hASCs undergoing adipogenic differentiation
ALP	Alkaline phosphatase
AM	Adipogenic medium
C/EBPβ	CCAAT/enhancer binding protein beta
CCK-8	Cell counting kit-8
Cd-ASC	hASCs undergoing chondrogenic differentiation
CM	Chondrogenic medium
Col I	Type I collagen
Col II	Type II collagen
Col X	Type X collagen
DEX	Dexamethasone
DQ-BSA	Dye quenched-bovine serum albumin
ECM	Extracellular matrix
GAG	Glycosminoglycan
hASCs	Human adipose-derived stem cells
MMP-13	Matrix metalloproteinase 13
MMPs	Matrix metalloproteinases
MSCs	Mesenchymal stem cells
Od-ASC	hASCs undergoing osteogenic differentiation
OM	Osteogenic medium
PPARγ	Peroxisome proliferator activated receptor gamma
RPS18	Ribosomal protein S18
RUNX2	Runt-related transcription factor 2
Sox9	Sex determining region Y-box9
TGF	Transforming growth factor
Ud-ASC	Undifferentiating hASCs
